# The intracerebral injection of Aβ_1-42_ oligomers does not invariably alter seizure susceptibility in mice

**DOI:** 10.3389/fnagi.2023.1239140

**Published:** 2023-09-07

**Authors:** Maxime Vande Vyver, Louise Daeninck, Gino De Smet, Najat Aourz, Surajit Sahu, Sebastiaan Engelborghs, Kris Pauwels, Dimitri De Bundel, Ilse Smolders

**Affiliations:** ^1^Department of Pharmaceutical Chemistry, Drug Analysis and Drug Information, Research Group Experimental Pharmacology (EFAR), Center for Neurosciences (C4N), Vrije Universiteit Brussel, Brussels, Belgium; ^2^Department of Neurology and Bru-BRAIN, Universitair Ziekenhuis Brussel, Brussels, Belgium; ^3^NEUR Research Group, Center for Neurosciences (C4N), Vrije Universiteit Brussel, Brussels, Belgium; ^4^Department of Biomedical Sciences, Reference Center for Biological Markers of Dementia (BIODEM), University of Antwerp, Antwerp, Belgium; ^5^RESEARCH Department, Vrije Universiteit Brussel, Brussels, Belgium

**Keywords:** amyloid beta 1-42, oligomer, Alzheimer’s disease, epilepsy, seizure

## Abstract

**Objectives:**

Epileptiform activity and seizures are present in patients with Alzheimer’s disease (AD) and genetic animal models of AD. Amyloid beta 1-42 (Aβ_1-42_) oligomers are thought to be crucial in AD and can cause neuronal hyperexcitability *in vitro*. However, it is unclear whether these Aβ_1-42_ oligomers cause the increased seizure susceptibility *in vivo* in people with AD and in AD animal models, nor via which mechanisms it would do so. We investigated this question by injecting Aβ_1-42_ oligomers intracerebrally in mice and assessed its impact on seizure susceptibility.

**Materials and methods:**

We performed a single intracerebral injection of synthetic Aβ_1-42_ oligomers or scrambled Aβ_1-42_ in NMRI mice in three different cohorts and subjected them to an i.v. infusion of a chemoconvulsant. We evoked the seizures 1.5 h, 1 week, or 3 weeks after the intracerebral injection of Aβ_1-42_ oligomers, covering also the timepoints and injection locations that were used by others in similar experimental set-ups.

**Results:**

With a thioflavine T assay and transmission electron microscopy we confirmed that Aβ_1-42_ monomers spontaneously aggregated to oligomers. We did not find an effect of Aβ_1-42_ oligomers on susceptibility to seizures – evoked 1.5 h, 1 week or 3 weeks – after their intracerebral injection.

**Significance:**

The lack of effect of Aβ_1-42_ oligomers on seizure susceptibility in our experiments contrasts with recent findings in similar experimental set-ups. Contradicting conclusions are frequent in experiments with Aβ_1-42_ and they are often attributed to subtle differences in the various aggregation forms of the Aβ_1-42_ used in different experiments. We confirmed the presence of Aβ_1-42_ oligomers with state-of-the-art methods but cannot ascertain that the protein aggregates we used are identical to those used by others. Whether our findings or those previously published best represent the role of Aβ_1-42_ oligomers on seizures in AD remains unclear.

## 1. Introduction

Patients with Alzheimer’s disease (AD) have an increased risk of developing seizures and epilepsy (Subota et al., 2017). One of the hallmarks of AD is the accumulation in the brain of amyloid beta (Aβ), a breakdown product of amyloid precursor protein (*APP*) (Jack et al., 2018). Several mouse models with an *APP* transgene mimic this Aβ accumulation. These mouse models also exhibit epileptic activity on EEG, are more susceptible to evoked seizures, and can even have spontaneous seizures (Ziyatdinova et al., 2011; Sanchez et al., 2012; Johnson et al., 2020; Vande Vyver et al., 2022). Whether it is *APP* or one of its breakdown products that causes this increased neuronal activity and seizure phenotype in genetic mouse models of AD is still subject to debate, although the main suspect is Aβ (Vogt et al., 2011; Born et al., 2014; Mensch et al., 2021).

We and others showed that Aβ plaques are not required for increased seizure susceptibility in AD mouse models (Bezzina et al., 2015; Vande Vyver et al., 2022). This means that the culprit should be sought upstream in the Aβ cascade. Monomeric Aβ spontaneously aggregates into oligomers and the most aggregation-prone subtype of Aβ is 42 amino acids long (Aβ_1-42_). Aβ_1-42_ oligomers are thought to be the aggregation form that increases neuronal activity the most (Hector and Brouillette, 2021). Incubating hippocampal slices for 2 h in Aβ_1-42_ oligomers increased the firing rate of pyramidal neurons and excitatory post synaptic potentials (Bao et al., 2021). In neuronal cultures, adding Aβ_1-42_ oligomers increases Na^+^ currents measured with patch-clamping, with a maximal effect after 24 h (Ciccone et al., 2019). One must differentiate data demonstrating neuronal hyperactivity in cultures or slices by Aβ_1-42_ oligomers from ictogenic mechanism: first, seizures result from a complex interplay between excitatory and inhibitory neurons; second, neuronal synchronization is more important than neuronal activity *per se* in seizures; and third, glial cells play a major role in ictogenesis (Blauwblomme et al., 2014; Hansen et al., 2018; Hiragi et al., 2018; Dejakaisaya et al., 2021).

Attempting to identify the specific culprit for increased seizure susceptibility in the *APP* cascade by using genetic mouse models of AD is notoriously difficult, since inhibiting the production of one breakdown product results in the accumulation of peptides of other processing pathways that might also impact neuronal activity. In addition, because of the relatively long interval between the genetic manipulation and the seizure readout in these genetic mouse models (3 weeks prenatal plus at least 3 weeks postnatal), it is also unclear whether the increased seizure susceptibility results from a direct effect of *APP*/Aβ (e.g., interaction with a receptor or formation of a membrane ion channel by Aβ) or from downstream events such as alterations in protein translation, inflammation, blood–brain barrier disruption, or cellular death. These phenomena are all present in AD and genetic mouse models of AD, and have the potential to impact seizure susceptibility (Blauwblomme et al., 2014; Klein et al., 2018; González et al., 2019; Dejakaisaya et al., 2021).

We thus aimed to assess the effect of Aβ_1-42_ oligomers on seizure susceptibility in a more direct way, by injecting Aβ_1-42_ oligomers intracerebrally and eliciting a seizure by administering a chemoconvulsant. This allows to control both the peptide of interest and the time during which the peptide could exert its effect.

While performing our experiments, two papers that assessed the effect of intracerebral injections of Aβ_1-42_ on seizure susceptibility in rodents were published. Alcantara-Gonzalez and colleagues found that 3 weeks after the intracerebroventricular (i.c.v.) injection of Aβ_1-42_ oligomers, rats were more susceptible to seizures induced by the K^+^ channel blocker 4-aminopiridine (4AP) (Alcantara-Gonzalez et al., 2019). Earlier in 2023, Bellingacci and colleagues showed that 1 week after injecting Aβ_1-42_ oligomers in the dentate gyrus (DG), the susceptibility to seizures evoked by the gamma-aminobutyric acid type A (GABA_A_) receptor antagonist bicuculline or 4AP was increased in mice (Bellingacci et al., 2022).

In our initial cohort we used the same quantity of Aβ_1-42_, Aβ_1-42_ oligomer preparation, and injection location as Brouillette and colleagues, who showed that these injections affected cognition in mice (Brouillette et al., 2012). One week after injecting Aβ_1-42_ oligomers into the DG, the gateway to the hippocampus, we assessed seizure susceptibility in these mice with the GABA_A_ receptor antagonist pentylenetetrazole (PTZ). We performed a second experiment, in which we shortened the interval between Aβ_1-42_ oligomer injection and the induction of a seizure to 1.5 h. Since the interval between intracerebral injection and the evoked seizure is short, we hypothesized that Aβ_1-42_ oligomers would exert its effect locally. We so chose kainic acid (KA) as chemoconvulsant, since it is the archetype of a chemoconvulsant eliciting seizures originating in the hippocampus. Lastly, we also performed an experiment to replicate the findings of Alcantara-Gonzalez and colleagues.

## 2. Materials and methods

### 2.1. Aβ_1-42_ and scrambled Aβ_1-42_ monomerization

Aβ_1-42_ (A1163-2) and scrambled Aβ_1-42_ (A-1004-2) were purchased from rPeptide and monomerized as described previously (Broersen et al., 2011). First, 0.5 mL of hexafluoro-2-propanol (HFIP) was added to the vials containing the peptide. After vortexing for 30 s, HFIP was evaporated under a gentle stream of nitrogen gas. The DMSO-solubilized peptides were applied to a HiTrap desalting column (17-1,408-01, GE Healthcare) that was previously equilibrated with phosphate buffered saline (PBS) with 1 mM ethylenediaminetetraacetic acid (EDTA) and eluted with the same buffer to obtain a DMSO-free peptide solution. The Aβ_1-42_ or scrambled Aβ_1-42_ in PBS with 1 mM EDTA was stored in pre-cooled LoBind tubes (0030108442, Eppendorf) on ice and the peptide concentration was calculated via ultraviolet absorption at 280 nm with a Nanodrop (Nanodrop 2000, ThermoScientific). The peptide was then aliquoted in LoBind tubes, snap frozen in liquid nitrogen, and stored at −80°C until injection. The monomers were injected within 1 week after storage at −80°C.

### 2.2. Thioflavine T fluorescence assay

The thioflavine T (ThT) assay was used to measure β-sheet formation over time with a fluorescence read-out. Different concentrations (0, 10, 20, 100 μM) of Aβ_1-42_ and scrambled Aβ_1-42_ were added to non-binding 96-well microplates (655,906, Greiner) with 12 μM of ThT in PBS with 1 mM EDTA. Fluorescence was measured at 5 min-intervals for 8 h on a plate reader (Victor 31,420 Multilabel Counter, Perkin Elmer) at 21°C using excitation and emission wavelengths of 440 nm and 480 nm, respectively, with an automated protocol.

### 2.3. Transmission electron microscopy

Aβ_1-42_ was allowed to aggregate for different time intervals. A 4 μL aliquot of 100 μM Aβ_1-42_ in PBS 1 mM EDTA was absorbed on 150-mesh formvar coated cupper grids for 1 min before it was blotted. The grid was washed twice with milli-Q water, after which it was stained for 30 s with uranyl acetate 2% in veronal buffer. The grids were then washed four times with milli-Q water, after which they were allowed to dry and stored until imaging. Imaging was done with a Tecnai 10 Philips transmission electron microscope (TEM) at an operating voltage of 80 kV. Images were acquired with a mega viewG2 CCD camera (SIS-company) and visualized with iTEM software.

### 2.4. Mice

Six-week-old male NMRI mice were bought from Charles River (France). Mice were habituated for 1 week to our facility before starting the experiment and were group housed (4–5 mice per cage) for the entire experiment. The experimental procedures were approved by the ethical committee of the Vrije Universiteit Brussel (19-213-10 and 22-213-4). Both the ARRIVE guidelines and the Basel declaration were considered when designing the experiments.

### 2.5. Stereotaxic injection

General anesthesia was induced with 4% isoflurane in an induction chamber for 2 min. Mice were then fixed on a stereotaxic frame and the isoflurane concentration was reduced to 1–2% for the rest of the procedure. 5 mg/kg meloxicam (Metacam, Boehringer Ingelheim) and 1 mL NaCl 0.9% were administered subcutaneously. A 2 cm scalp incision was made, after which we verified skull flatness by ensuring that the dorsoventral (DV) deviation between bregma and +/− 1.00 mediolateral (ML) and lambda was less than 0.1 mm. We then drilled holes at −2.20 anteroposterior (AP) and +/− 1.40 ML for DG injection or at −0.34 AP and + 1.00 ML for the i.c.v. injection. The dura mater was punctured with a 29G needle and the microsyringe (700/1700 series 65,460–05, Hamilton) was slowly advanced through the drill holes to −2.10 DV for DG injection and − 2.70 DV for i.c.v. injection. The 100 μM Aβ_1-42_ or 100 μM scrambled Aβ_1-42_ in PBS with 1 mM EDTA was previously allowed to oligomerize for 1.5–2 h at room temperature. Mice in which we induced a seizure 1 week after Aβ_1-42_ injection received a 1 μL (0.2 μL/min) injection in both DG. A 2 μL injection (0.4 μL/min) was performed in both DG of mice in which we evoked a seizure 1.5 h after Aβ_1-42_ injection. Mice that were subjected to a seizure 3 weeks after the injection of Aβ_1-42_ received a unilateral i.c.v. injection of 10 μL (2 μL/min). After injection, the microsyringe was left in place for 5 min, after which it was slowly taken out and the skin sutured. Finally, mice were allowed to recover in a heated recovery chamber until they regained full mobility. The duration of anesthesia ranged between 30 and 40 min.

### 2.6. i.v. seizure models

Seizures were evoked by a continuous i.v. infusion (150 μL/min) of a chemoconvulsant in the lateral tail vein, as we previously published (Schallier et al., 2009; Portelli et al., 2012). We diluted 7.5 mg/mL KA, 7.5 mg/mL PTZ, or 4 mg/mL 4AP in 0.9% NaCl with 10 IU/mL heparin. Mice were put in a restrainer and the tail was briefly warmed in water at 37°C, after which the lateral tail vein was punctured with a 30G needle. The infusion pump was started to ensure correct i.v. delivery of the chemoconvulsant and the needle was fixed with tape to the tail. PTZ infusion resulted in (1) myoclonic twitch, (2) Straub tail, (3) forelimb clonus, (4) falling, (5) THE, and (6) death. KA-induced seizures resulted in the following phenotype: (1) behavioral arrest, (2) falling, (3) tonic hindlimb extension (THE), and (4) death. 4AP infusion resulted in (1) eye blinking, (2) jumping, (3) THE, and (4) death. Mice were then taken out of the restrainer into a transparent cage to assess the different seizure stages. Unsuccessful i.v. delivery of the infusion resulted in whitening of the tail and absence of seizure stages. These mice were discarded from the analysis. Mice were videotaped and the occurrence of seizures stages was evaluated by experimenters blinded to the test groups. The chemoconvulsant dose was calculated as follows:

$$infusiontime\;\left(s\right)\times infusionspeed\;\left(\frac{ml}{s}\right)\;dose\;\left(\frac{mg}{kg}\right)=\frac{\times \left[chemoconvulsant\right]\;\left(\frac{mg}{ml}\right)}{bodyweight\;\left(kg\right)}$$

### 2.7. Statistics

The statistical analysis was performed with Rstudio (2022.02.0). The results of i.v. seizure models were assessed with linear mixed-effects models in which the seizure stages and the injected peptide were fixed effects and the sequential stages within a mouse a random effect: dose ∼ seizure stage * injected peptide + (1|id). Normality of data was assessed with a qqplot, variance with a fitted plot. The evolution of the seizure stages was not linear over time, so we analyzed the different seizure stages as an ordinal categoric value, not as a numeric one. If a significant effect between groups or an interaction between seizure stage and group was present, multiple comparisons were assessed with Tukey’s test. When there was no interaction between seizure stage and the injected peptide, we simplified the model to: dose ∼ seizure stage + injected peptide + (1|id). Data were reported as mean ± standard deviation. α was set at 0.05.

## 3. Results

### 3.1. Characterization of Aβ_1-42_ aggregation *in vitro,* its effect in slices, and its intracerebral injection *in vivo*

**Figure 1:**
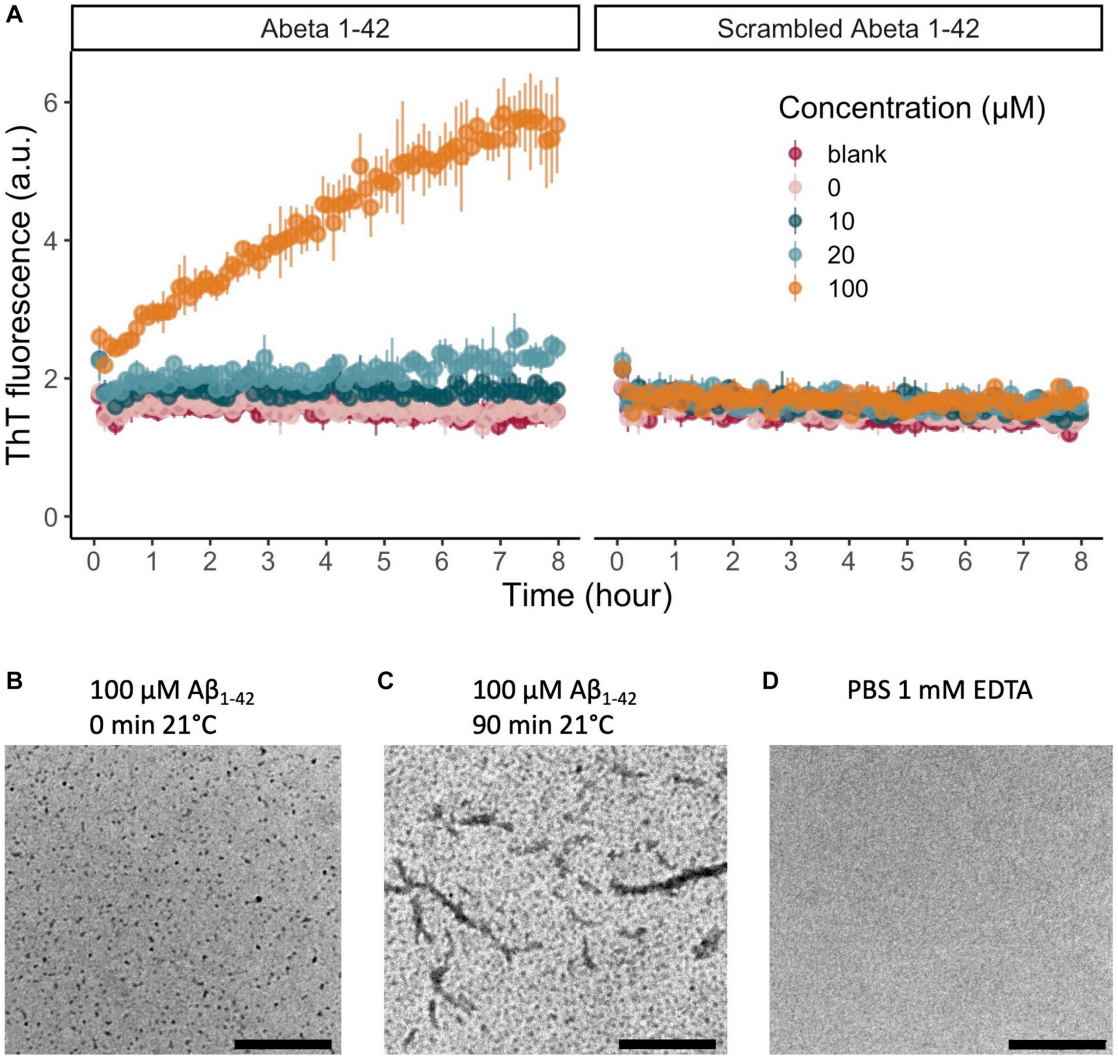
*In vitro* evaluation of the peptide solubilization procedure and aggregation properties of Aβ_1-42_. **(A)** Aβ_1-42_ aggregates over time and forms β-sheets that increase the fluorescence emission of ThT at 480 nm. We represented the data as mean ± standard deviation. There is a clear concentration-dependent increase in fluorescence of Aβ_1-42_ over time, indicating the aggregation of Aβ_1-42_ monomers to form β-sheets (*n* = 3 per concentration). As expected, there is no increase in fluorescent signal for scrambled Aβ_1-42_, that does not aggregate or form β-sheets (*n* = 3 per concentration). **(B)** After solubilization of Aβ_1-42_, monomers and small oligomers are present. **(C)** After 90 min of incubation at 21°C, TEM reveals that next to monomers and oligomers also protofibrils can be detected. **(D)** A TEM image of PBS 1 mM EDTA showed no staining. Scale bars represent 2.5 μm. (TEM, transmission electron microscopy; ThT, thioflavin T).

We verified the successful solubilization and monomerization of Aβ_1-42_ by characterizing the aggregation kinetics at 21°C with a ThT assay and TEM. The presence of a lag phase in the lower peptide concentrations (10 and 20 μM Aβ) in the ThT curve confirmed the monomeric state of our starting material, while the increase in ThT fluorescence indicates the formation of β-sheets, the main secondary structure of Aβ_1-42_ oligomers and fibrils. ThT fluorescence increased gradually in a concentration-dependent manner in Aβ_1-42_ over the 8 h of measurement (Figure 1A) (*n* = 3 technical replicates). No increase in fluorescence was noted in any of the concentrations of scrambled Aβ_1-42_ (*n* = 3 technical replicates; Figure 1A). In addition to this biophysical measurement, we also visualized Aβ_1-42_ aggregation with TEM. We confirmed that the 100 μM Aβ_1-42_ solution oligomerized over time (Figures 1B,C). A TEM with PBS served as negative control (Figure 1D). These biophysical measurements confirm that we can replicate the standardized solubilization and aggregation of Aβ_1-42_ that was previously described (Kuperstein et al., 2010; Broersen et al., 2011). To assess the biologic effect of Aβ_1-42_ oligomers, we examined the intrinsic excitability of granular cells of the dentate gyrus in acute hippocampal slices from 6-week-old mice. After whole-cell patch-clamping these neurons, we looked at neuronal excitability before and after incubation of the slices in 500 nM Aβ_1-42_ oligomers for 20 min (Supplementary Figure S1). As expected, increasing the injected current augmented the number of fired action potentials (*F*_(1,207)_ = 325, *p* < 0.001). Compared to baseline, the number of action potentials elicited by a 70 pA current injection was higher after incubation of the slices in Aβ_1-42_ oligomers (*F*_(1,207)_ = 17, *p* < 0.001). There was a significant interaction between the injected current and the baseline or Aβ_1-42_ oligomer condition (*F*_(1,207)_ = 6, *p* = 0.1). In addition to the *in vitro* characterization, this demonstrates a biologic effect of our Aβ_1-42_ oligomers on neuronal excitability in acute hippocampal slices. Lastly, we validated successful Aβ_1-42_ injection and correct location of injection in the DG with an anti-Aβ_1-42_ DAB staining (Supplementary Figure S2).

### 3.2. There is no effect on seizure susceptibility one week after the injection of Aβ_1-42_ oligomers in the DG

**Figure 2:**
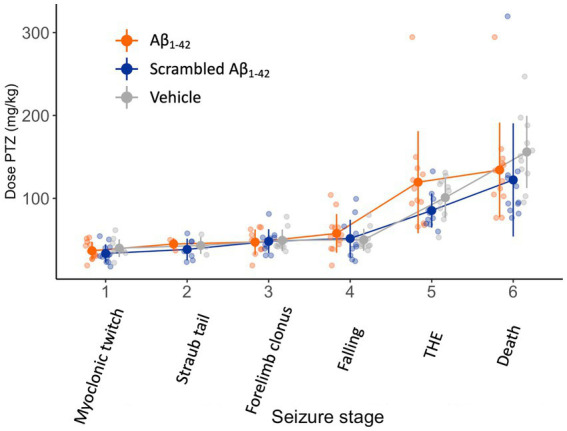
Seizure susceptibility is not affected 1 week after the injection of Aβ_1-42_ oligomers, scrambled Aβ_1-42_, or vehicle in the DG. NMRI mice received a bilateral stereotaxic injection of 1 μL 100 μM Aβ_1-42_ oligomers, 1 μL 100 μM scrambled Aβ_1-42_, or 1 μL vehicle in the DG (*n* = 11–14 per group). One week later, they were subjected to a continuous i.v. infusion of PTZ. This infusion results in progressively worsening seizures and death within a few minutes. There was no significant difference in dose required to attain any seizure stage between the three injection groups (*F*_(2,29)_ = 1, *p* = 0.4). Individual data points (semitransparent) and mean ± standard deviation (opaque) are represented. A small horizontal shift per group was added to the data points to improve readability. (DG, dentate gyrus; PTZ, pentylenetetrazole; THE, tonic hindlimb extension).

We first investigated seizure susceptibility 1 week after the injection of Aβ_1-42_ oligomers. As chemoconvulsant we used PTZ, a GABA_A_-antagonist similar to bicuculline used by Bellugacci and colleagues. The injection of Aβ_1-42_ was performed in the DG, the gateway to the large hippocampal neuronal pathways. In addition to scrambled Aβ_1-42_ as our preferred control, we also included a control group that was injected with vehicle. This allows us to discard the possibility that any injected peptide would have impacted seizure susceptibility and not Aβ_1-42_ or scrambled Aβ_1-42_ specifically. The continuous i.v. PTZ infusion resulted in progressively worsening seizures (*F*_(5,141)_ = 62, *p* < 0.001) (Figure 2). Injecting Aβ_1-42_, scrambled Aβ_1-42_, or vehicle did not impact the required dose to reach any of the seizure stages (*F*_(2,29)_ = 1, *p* = 0.4). To confirm that the i.v. tail infusion of PTZ can detect the effect of an intracerebral injection of a proconvulsant, we set up a very similar experiment in a small group of mice (*n* = 6 per group). In these mice, we replaced the injection of Aβ_1-42_ oligomers by a single unilateral injection of KA, a well-known chemoconvulsant, or vehicle in the DG. One week later, we performed an i.v. infusion of PTZ (Supplementary Figure S3). The infusion of PTZ resulted in progressively worsening seizures (*F*_(5,46)_ = 53, *p* < 0.001). There was no difference between groups in the dose required to reach seizure stage 1 (*F*_(1,10)_ = 4, *p* = 0.09), but there was a significant interaction between seizure stage and group (*F*_(5,46)_ = 4, *p* = 0.005). *Post hoc* pairwise comparison demonstrated that the group that received a KA injection reached stage 5 (THE) and 6 (death) more rapidly compared to the injection of vehicle (*p* = 0.01 and *p* = 0.006 respectively).

### 3.3. Seizure susceptibility is not affected 1.5 h after the injection of Aβ_1-42_ oligomers in the DG

**Figure 3:**
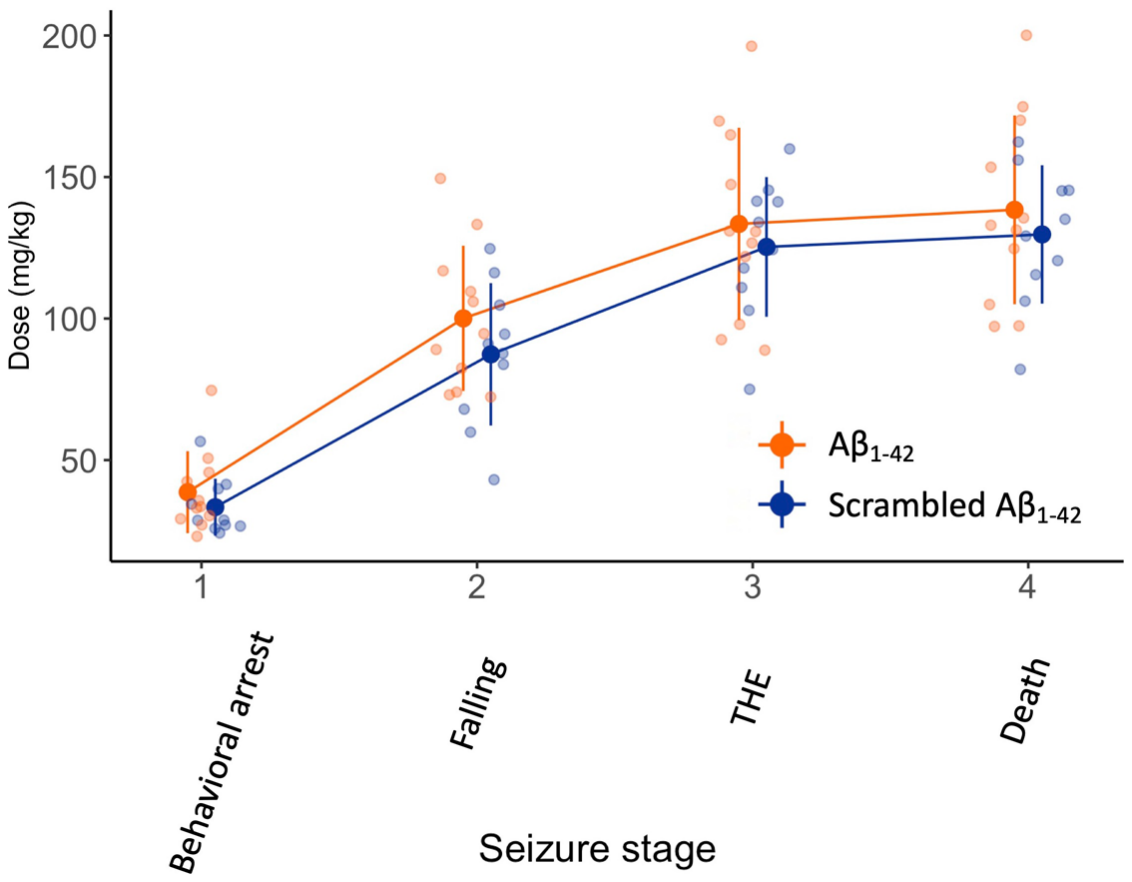
Ninety minutes after the injection of Aβ_1-42_ oligomers or scrambled Aβ_1-42_ in the DG seizure susceptibility is not affected. We injected 2 μL of 100 μM Aβ_1-42_ or 2 μL 100 μM scrambled Aβ_1-42_ that was previously allowed to oligomerized for 1.5–2 h at 21°C in the DG of NMRI mice (*n* = 9–11 per group). Ninety minutes later, we subjected the mice to a continuous i.v. infusion of KA that results in focal temporal seizures, evolving to a generalized seizure and eventually death. There was no significant difference in dose required to attain seizure stages between both groups (*F*_(1,19)_ = 1, *p* = 0.4). Individual data points (semitransparent) and mean ± standard deviation (opaque) are represented. A small horizontal shift per group was added to the data points to improve readability. (DG, dentate gyrus; KA, kainic acid; THE, tonic hindlimb extension).

We then assessed the effect of a bilateral Aβ_1-42_ oligomer injection in the DG on seizure susceptibility 1.5 h after injection. We chose this timeframe to keep the interval after injection as short as possible, still allowing mice to fully recover motor activity after anesthesia. As the seizure was evoked shortly after the Aβ_1-42_ injection, we chose KA as chemoconvulsant since KA elicits seizures originating in the hippocampus where Aβ_1-42_ was injected. The continuous infusion of KA resulted in gradually worsening seizures with focal temporal onset, progressing to bilateral motor seizures eventually resulting in death (*F*_(60,173)_ = 1, *p* < 0.001) (Figure 3). There was no difference between Aβ_1-42_ or scrambled Aβ_1-42_ in the dose required to attain the different seizure stages (*F*_(1,19)_ = 1, *p* = 0.4).

### 3.4. Three weeks after the i.c.v. injection of Aβ_1-42_ oligomers, seizure susceptibility is not affected

**Figure 4:**
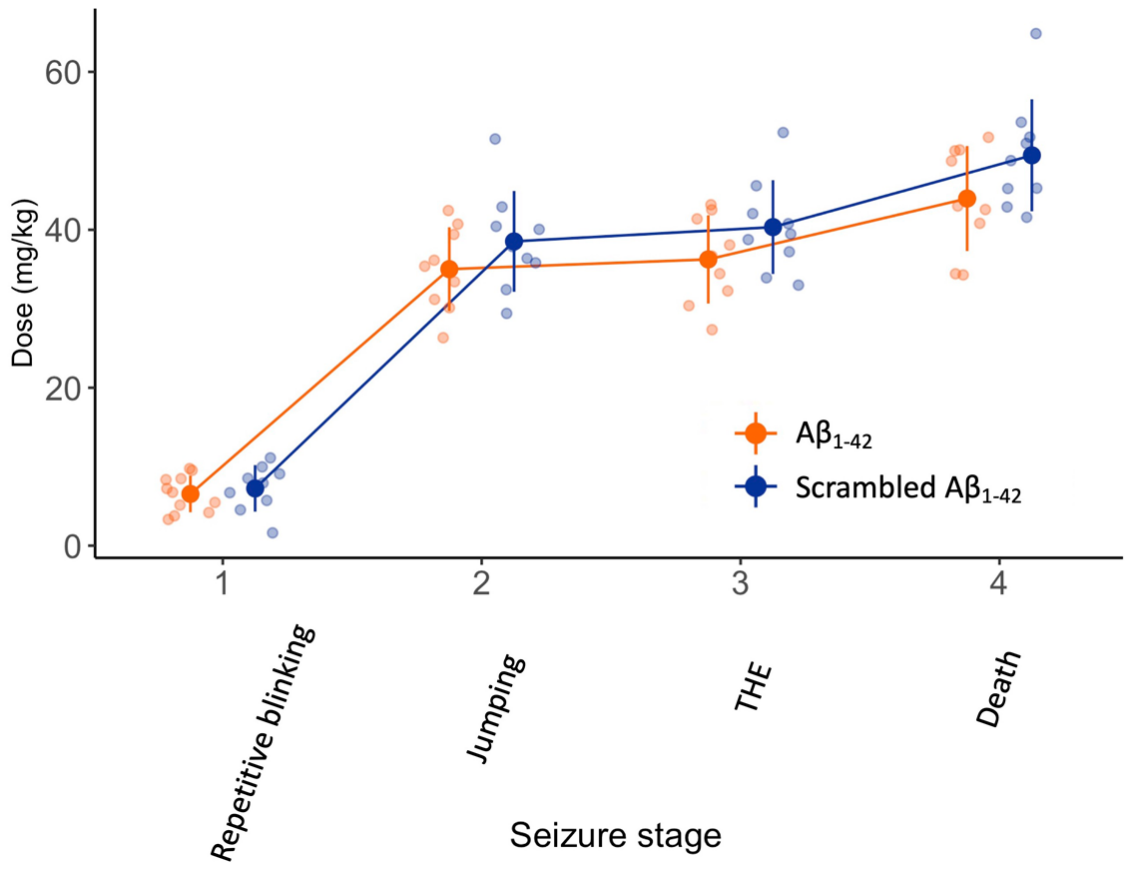
There is no difference in seizure susceptibility 3 weeks after intracerebroventricular injection of Aβ_1-42_ oligomers compared to scrambled Aβ_1-42_. NMRI mice received a 10 μL stereotaxic intracerebroventricular injection of 100 μM Aβ_1-42_ or 100 μM scrambled Aβ_1-42_ that was allowed to oligomerize for 1.5–2 h at 21°C (*n* = 9–11 per group). Three weeks later, they were subjected to a continuous i.v. infusion of 4AP. In both groups, the infusion resulted in a progressive seizure phenotype and eventually death. The dose of 4AP required to develop seizures stages was not different between mice injected with Aβ_1-42_ oligomers compared to scrambled Aβ_1-42_ (*F*_(1,18)_ = 3, *p* = 0.1). Individual data points (semitransparent) and mean ± standard deviation (opaque) are represented. A small horizontal shift per group was added to the data points to improve readability. (4AP, 4-aminopyridine; THE, tonic hindlimb extension).

Finally, we attempted to replicate the experiments of Alcantara-Gonzalez and colleagues. Three weeks after the injection of Aβ_1-42_ or scrambled Aβ_1-42_, we submitted the mice to an infusion of 4AP, a K^+^ channel blocker, as did Alcantara-Gonzalez and colleagues. The infusion resulted in progressively worsening seizures over time (*F*_(3,53)_ = 391, *p* < 0.001) (Figure 4). However, the dose of 4AP required to reach the seizure stages was not different between the mice injected with Aβ_1-42_ or scrambled Aβ_1-42_ (*F*_(1,18)_ = 3, *p* = 0.1).

## 4. Discussion

With these experiments, we assessed the impact of spontaneously formed Aβ_1-42_ oligomers on seizure susceptibility *in vivo* in mice. We were not able to detect a difference in seizure susceptibility or seizure development after the injection with Aβ_1-42_ oligomers or scrambled Aβ_1-42_ in any of our experimental set-ups. Since recently two other research teams demonstrated increased seizure susceptibility upon injection of Aβ_1-42_ in very similar experiments, we discuss the methodological differences between those studies and ours to explain the different results in detail.

### 4.1. What is the role of Aβ_1-42_ on seizure susceptibility?

The research interest in Aβ originally stems from the fact that it is the main constituent of Aβ plaques in AD patients. The focus on Aβ oligomers exploded after they were found to be synapto- and neurotoxic and that they correlated better with cognitive decline in mouse models than other factors in the Aβ cascade (Ferreira et al., 2015). Much of the scientific attention was drawn to Aβ_1-42_ as it is increased in people with autosomal dominantly inherited AD, aggregates rapidly due to its hydrophobicity, and reduces long term potentiation and synaptic density (Walsh et al., 2002; Shankar et al., 2008).

The effect of Aβ_1-42_ oligomers on neuronal activity has been extensively studied *in vitro*, with conclusions that are not completely congruent. In hippocampal slices, Aβ_1-42_ oligomers increased paired pulse ratios of population spikes in the DG, and increased the amplitude of population spikes in the presence of bicuculline in the DG (Costa et al., 2016). Also we showed that incubation of acute hippocampal slices in a solution with synthetic Aβ_1-42_ oligomers increased the neuronal excitability of granule cells of the DG. In pyramidal neurons of slices of the anterior cingulate cortex, Aβ_1-42_ oligomers increased the frequency of action potentials induced by current injections and decreased the frequency and amplitude of miniature inhibitory post-synaptic potentials (Ren et al., 2018). Intracellular infusion of Aβ_1-42_ oligomers, but not monomers, increased the α-amino-3-hydroxy-5-methyl-4-isoxazolepropionic acid (AMPA)-regulated excitatory post-synaptic currents within minutes (Whitcomb et al., 2015). The intracellular injection of Aβ_1-42_ oligomers increased miniature excitatory postsynaptic currents in primary neuronal cultures and increased the number of action potentials in *cornu ammonis* 1 neurons *in vivo* in anesthetized mice (Fernandez-Perez et al., 2021). However, another study showed that Aβ_1-42_ oligomers reduced neuronal activity *in vitro* in cultures measured with multiple electrode array (Kuperstein et al., 2010). In that same study Aβ_1-40_ on the other hand increased neuronal activity.

Besides Aβ_1-42_ oligomers, Aβ_1-40_ oligomers thus also received much attention. Aβ_1-40_ oligomers increased neuronal activity assessed with electrophysiology and calcium imaging in hippocampal cultures (Cuevas et al., 2011). Applying 500 nM Aβ_1-40_ dimers with a S26C mutation to the *cornu ammonis* 1, increased neuronal calcium transients within 15 min *in vivo* (Busche et al., 2012; Zott et al., 2019). The authors argued that the increase in neuronal activity required baseline activity, as in slices this effect was only present after increasing basal neuronal activity with bicuculline, glutamate, or increased K^+^ concentration. This again stresses that *in vitro* findings on neuronal activity cannot *per se* be translated *in vivo.* Moreover, Aβ_1-40_ application also resulted in reduced neuronal activity in neuronal cultures in a study (Sepúlveda et al., 2009). Some studies have even questioned the effect of Aβ in general on epileptiform activity *in vivo*. Johnson and colleagues assessed the effect of early and prolonged reduction of Aβ on epileptiform activity on EEG *in vivo* (Johnson et al., 2020). Reduction of Aβ (including Aβ oligomers) with a BACE inhibitor did not change epileptiform activity on EEG. The impact of Aβ oligomers on neuronal network excitability is thus still unclear.

What are the proposed mechanisms by which Aβ_1-42_ would lead to increased seizure susceptibility? The very rapid response *in vitro* would suggest a fast receptor-dependent mechanism. There is much evidence for a direct interaction of Aβ_1-42_ with N-methyl-D-aspartate, AMPA and GABA receptors (Ferreira et al., 2015; Palop and Mucke, 2016; Fernandez-Perez et al., 2021). Another frequently postulated rapid mechanism is that its hydrophobic properties allow Aβ_1-42_ to form cationic channels in neuronal membranes. Only Aβ_1-42_ oligomers, not Aβ_1-42_ monomers or Aβ_1-40_ oligomers, did so in the membrane of HEK293 cells (Bode et al., 2017). As Aβ_1-40_ also alters neuronal activity, it is improbable that the Aβ pores are the cause of the bulk of the biological mechanism. Aβ_1-42_ was proposed to exert its effect by increasing the release of glutamate by astrocytes in culture and reducing the inhibitory tone in slices and in mice (Ulrich, 2015; Sanz-Blasco et al., 2016; Calvo-Flores Guzmán et al., 2020). Pinpointing the exact mechanism is very difficult, as alterations in excitatory tone will immediately result in compensatory changes in the inhibitory tone and vice versa. As these compensatory mechanisms occur very rapidly, the chicken or egg question is very complex to answer experimentally.

### 4.2. What happens to Aβ_1-42_ after injection into the DG or i.c.v.?

Both Aβ_1-42_ injections in the DG and i.c.v. have been frequently used as experimental models and were shown to be neurotoxic and result in cognitive impairments in rodents (Chambon et al., 2011). Aβ_1-42_ oligomers (but not fibrils) penetrate from the cerebrospinal fluid into the brain parenchyma via the ventricular wall within 5 min (Kasza et al., 2017). Although we attempt to make our procedures minimally invasive, the ventricular wall will be damaged from the i.c.v. injection, further facilitating the entry of Aβ_1-42_ oligomers into the brain parenchyma. It is unclear if the effect of an injection of Aβ_1-42_ oligomers into the DG results from a local effect in the DG or if Aβ_1-42_ oligomers need to diffuse. After a single injection of Aβ_1-42_ oligomers in the DG, Aβ_1-42_ is no longer detectable at the DG after a few days (Brouillette et al., 2012). Still, several studies found cognitive impairments in mice several weeks after a single injection of Aβ_1-42_ (Chambon et al., 2011; Karthick et al., 2018). It is possible that Aβ_1-42_ oligomers resulted in structural damage (although Brouillette and colleagues did not detect neuronal death) or that it initiated downstream events that exert their effect later on, independent of Aβ_1-42_. Aβ_1-42_ oligomers would be the match that ignites the fire that can then rage out of control afterwards. An interesting approach that could unravel the changes induced by Aβ_1-42_ oligomers in these local injection models would be to perform “-omics” on punch biopsies of the DG region at different intervals post injection.

### 4.3. Is all Aβ_1-42_ created equal?

The next question is how much Aβ_1-42_ one should inject to create these models. It is important to first consider the origin of the injected Aβ_1-42_. Chemically synthesized Aβ_1-42_ requires a much higher concentration to exert a similar biological effect compared to human-derived Aβ_1-42_ from brain samples of patients with AD (Varshavskaya et al., 2022). These varying biological effects probably result from differences in aggregation dynamics. Indeed, subtle changes in medium may affect, not only the speed of aggregation, but even guide Aβ_1-42_ to a very different aggregation end product (Brody et al., 2017; McAllister et al., 2020). Even within patient-derived Aβ preparations there are big differences. For example, Aβ_1-42_ from brain homogenates are potent seeds for Aβ plaques, whereas Aβ_1-42_ from human cerebrospinal fluid has no seeding capacity (Fritschi et al., 2014). We used a very similar methodology as Brouillette and colleagues to generate our Aβ_1-42_ with the same Aβ_1-42_ manufacturer, an identical monomerization process and aggregation time, and equivalent amount of moles of injected Aβ_1-42_ (Brouillette et al., 2012). Their Aβ_1-42_ preparation induced memory deficits and neurotoxicity. We validated the aggregation state of our Aβ_1-42_ oligomers with two performant techniques of which the results were perfectly in line with previously published methodologic papers and ensured that we injected the Aβ_1-42_ oligomers at the correct location (Broersen et al., 2011). Validating the biophysical properties of the used Aβ_1-42_ preparations is crucial, because of the well-known complex behavior of this sticky and aggregating peptide that is sensitive to the smallest changes. In addition, we demonstrated that the Aβ_1-42_ oligomers have a biologic effect on neuronal excitability of granular cells of the dentate gyrus in acute slice electrophysiology.

Knowing whether the amount of Aβ_1-42_ oligomers we injected is similar to the concentration in AD patients is impossible. One would have to know the concentration of Aβ_1-42_ intracellularly and in the interstitial fluid of patients, consider regional differences of Aβ concentration in the brain, have a precise knowledge of the fraction of each aggregation form, know the diffusion dynamics and half-life of Aβ_1-42_ oligomers, and know which Aβ_1-42_ oligomer pools are relevant to its (patho)biologic effect (Cirrito et al., 2003; Koffie et al., 2009; Keskin et al., 2017). The dose we used for i.c.v. injection was identical to that of Alcantara-Gonzalez and colleagues and so was the dose we injected in the DG for the mice subjected to a seizure 1.5 h later. For the mice that were subjected to a seizure 1 week after Aβ_1-42_ injection in the DG, we used the dose of Brouillette and colleagues, which is only half the dose of Bellingacci and colleagues (see Supplementary Table S1 for an overview) (Brouillette et al., 2012; Bellingacci et al., 2022). It is unclear if this two-fold difference is relevant in a context where doses of Aβ_1-42_ oligomers of different orders of magnitude all showed an effect (Berry et al., 2018). Compared to Bellingacci and colleagues, we also reduced the stress that the injection causes on the DG brain tissue by splitting our injection over both hemispheres.

### 4.4. How to choose the chemoconvulsant, control injection, and rodent model?

Acute seizures elicited by chemoconvulsants (frequently PTZ and KA) successfully demonstrated increased susceptibility in genetic AD mouse models (Jolas et al., 2002; Del Vecchio et al., 2004; Palop et al., 2007). KA is an agonist of glutamatergic KA-receptors. We chose this chemoconvulsant to induce seizures rapidly after injection into the DG because KA results in seizures with hippocampal onset (Coppola and Moshé, 2012). As for both experiments with delayed seizures, in which the Aβ_1-42_ already had the occasion to diffuse, we used PTZ and 4AP that model generalized seizures as did Alcantara-Gonzalez and colleagues and Bellingacci and colleagues. The experiment is based on the “two-hit” model of seizures in which a first epileptogenic substance is injected into the brain, followed by a different method to elicit the seizure. This was first described with an injection of KA into the hippocampus, followed by amygdala kindling. The group of rats that received a KA injection first, developed seizures much quicker (Feldblum and Ackermann, 1987). This two-hit phenomenon is not limited to classic chemoconvulsants. Increasing or decreasing the concentration in the hippocampus of molecules that affect general excitability can also affect seizure susceptibility in this two-hit paradigm (Vezzani et al., 1999; Kelley et al., 2018).

The two above mentioned studies that assessed the effect of intracerebral Aβ_1-42_ injection on seizure susceptibility used vehicle as control (Alcantara-Gonzalez et al., 2019; Bellingacci et al., 2022). Since various peptides alter seizure susceptibility, we used both a vehicle and a scrambled Aβ_1-42_ control group to rule out this possibility (Clynen et al., 2014; Manavi, 2022). Our data suggest that the effects they found would not have been induced by any peptide, as our scrambled Aβ_1-42_ and vehicle groups did not differ in seizure susceptibility. However, even more crucially, their Aβ_1-42_ solution contained 2% DMSO, which was not present in any of their vehicle groups. As DMSO impacts seizure susceptibility *in vivo*, this seems a potentially crucial difference (Bauwens et al., 2005; Kovács et al., 2011).

We used a different species than Alcantara-Gonzalez and colleagues, and a different mouse strain than Bellingacci and colleagues, who used outbred Wistar rats and inbred C57/Bl6 mice, respectively. We deliberately opted for outbred mice, as their genetic heterogeneity better represents a real-world situation. It is well-known that genetic background affects susceptibility to seizures, both in wildtype mice and AD mouse models (Jackson et al., 2015; Leclercq and Kaminski, 2015). The genetic background of AD mouse models also affects their Aβ dynamics (Jackson et al., 2015). However, it is difficult to attribute the contrasting conclusions between our studies only by the different species and strain we used since both a study on inbred mice and a study on outbred rats detected an increased seizure susceptibility after Aβ_1-42_ oligomer injection.

The major limitation of our study is inherent to the fact that we were not able to demonstrate any difference between the Aβ_1-42_ and scrambled Aβ_1-42_ groups *in vivo*. However, we showed that the i.v. PTZ tail infusion seizure model is able to detect the effect of a single intracerebral injection of the established proconvulsant KA under similar experimental conditions. In addition, we showed that a 20 min incubation of acute hippocampal slices in 500 nM Aβ_1-42_ oligomers increased the number of action potentials fired by patched granular cells of the DG. This way, we confirmed the sensitivity of the seizure model *in vivo* and a biological action of our Aβ_1-42_ oligomer preparation *ex vivo* in slices. A *post hoc* power calculation of the Aβ_1-42_ experiments only has limited value. When comparing our sample sizes to the two papers that demonstrated an effect of Aβ_1-42_ oligomer injection on seizure susceptibility, we have more data points per group than Alcantara-Gonzalez and colleagues, but less than Bellingacci and colleagues.

As discussed throughout this paper, the effect of Aβ varies greatly in literature and probably depends on multiple factors that are not yet considered or cannot be controlled for. Publishing these results that contradict earlier work is key, especially in the field of Aβ_1-42_ in which conclusions diverge much between studies. This again stresses the importance of thorough biophysical characterization of the used Aβ_1-42_ preparation, to allow comparison between studies. Only the publication of experiments in which significant effects are found, and those in which they aren’t, can provide readers with the most complete representation of scientific findings. Here we can only deduce that earlier seizure threshold lowering effects of Aβ_1-42_ cannot be generalized to all seizure models and experimental conditions.

In conclusion, we did not find an increased seizure susceptibility in mice injected with Aβ_1-42_. This contrasts with two other published reports. Our biophysical validation of Aβ_1-42_ oligomers was however state-of-the-art, we demonstrated its effects on *ex vivo* neuronal excitability and showed successful *in vivo* intracerebral Aβ_1-42_ injection in mice.

## Data availability statement

The raw data supporting the conclusions of this article will be made available by the authors, without undue reservation.

## Ethics statement

The animal study was approved by Ethische Commissie Dierproeven Vrije Universiteit Brussel. The study was conducted in accordance with the local legislation and institutional requirements.

## Author contributions

MV, KP, and IS conceptualized and designed the study. Study design was further adapted with valuable suggestions from NA, SS, SE, and DB. MV, LD, GS, NA, and SS performed the experiments. MV analyzed the data. MV and IS drafted the manuscript. MV, LD, GS, NA, SS, SE, KP, DB, and IS reviewed the manuscript. All authors contributed to the article and approved the submitted version.

## Funding

This work was supported by grants of FWO Research Foundation – Flanders (11E4819N and G040419N) and Wetenschappelijk Fonds Willy Gepts of Universitair Ziekenhuis Brussel.

## Conflict of interest

The authors declare that the research was conducted in the absence of any commercial or financial relationships that could be construed as a potential conflict of interest.

## Publisher’s note

All claims expressed in this article are solely those of the authors and do not necessarily represent those of their affiliated organizations, or those of the publisher, the editors and the reviewers. Any product that may be evaluated in this article, or claim that may be made by its manufacturer, is not guaranteed or endorsed by the publisher.
