# Supplementary material for: The intracerebral injection of Aβ1-42 oligomers does not invariably alter seizure susceptibility in mice
Source: Front Aging Neurosci. 2023 Sep 7;15:1239140. doi: 10.3389/fnagi.2023.1239140 (PMC10512828; doi:10.3389/fnagi.2023.1239140)
Supplement: Supplementary file 1 [file Data_Sheet_1.PDF]

The intracerebral injection of Aβ1-42 oligomers does not invariably alter seizure susceptibility in mice

Supplementary information

Maxime Vande Vyver[1,2,3,4], Louise Daeninck[1], Gino De Smet[1], Najat Aourz[1], Surajit Sahu[1], Sebastiaan Engelborghs[2,3,4], Kris Pauwels[5], Dimitri De Bundel[1], Ilse Smolders[1*]

[1]Department of Pharmaceutical Chemistry, Drug Analysis and Drug Information, Research Group Experimental Pharmacology (EFAR), Center for Neurosciences (C4N), Vrije Universiteit Brussel, Brussels, Belgium

[2]Department of Neurology and Bru-BRAIN, Universitair Ziekenhuis Brussel, Brussels, Belgium

[3]NEUR Research Group, Center for Neurosciences (C4N), Vrije Universiteit Brussel, Brussels, Belgium

[4]Reference Centre for Biological Markers of Dementia (BIODEM), Department of Biomedical Sciences, University of Antwerp, Antwerp, Belgium

[5]RESEARCH department, Vrije Universiteit Brussel, Brussels, Belgium

*** Correspondence:**
Prof. Dr. Ilse Smolders
Ilse.Smolders@vub.be

Keywords: Amyloid beta 1-42, oligomer, Alzheimer's disease, Epilepsy, Seizure

1. Supplementary materials and methods

1.1 *Ex vivo* electrophysiology in hippocampal slices

Coronal slices (300 µm) from six-week-old mice were prepared with a VT 1000S vibratome (Leica) in oxygenated ice-cold high-choline artificial cerebrospinal fluid (ACSF) containing (in mM): 130 choline; 2.5 KCl; 1.25 NaH_2PO_4, H_2O; 7 $MgCl_2$, 0.5 $CaCl_2$, $6H_2O$; 25 $NaHCO_3$ and 7 glucose at 4-6 °C. Slices were then maintained at room temperature in oxygenated ACSF containing (in mM): 126 NaCl; 3.5 KCl; 1.2 NaH_2PO_4, H_2O; 1.3 $MgCl_2$; 2 $CaCl_2$, $6H_2O$; 25 $NaHCO_3$ and 11 glucose. Slices were then transferred one at a time to a submersion recording chamber and were perfused continuously with oxygenated ACSF warmed to 31 °C at a rate of 2–2.5 ml/min. All solutions were equilibrated with 95% O_2/5% CO_2. Neurons were visualized on an upright microscope (Scientifica SliceScope Pro 6000) equipped with DIC optic and filter set to visualize cells using a x40 water-immersion objective. To measure intrinsic properties of the DG granule cells, we used current-clamp recordings. Glass electrodes (6–8 MΩ) were filled with an internal solution containing the following (mM): 130 $KMeSO_4$; 5 KCl; 10 4-(2-hydroxyethyl)-1-piperazineethanesulfonic acid; 2.5 MgATP; 0.3 NaGTP; 0.2 ethyleneglycoltetraacetic acid; and 10 phosphocreatine, pH = 7.21. Access resistance ranged between 20 and 30 MΩ, and the results were discarded if the access resistance changed by >20%. Data were collected with a MultiClamp 700B amplifier (Molecular Devices), filtered (3 kHz) and digitized (20 kHz) with a Digidata 1440 A (Molecular Devices) to a personal computer, and acquired using Clampex 10.1 software (PClamp, Axon Instruments, Molecular Devices). Data were analyzed in clampfit (Molecular Devices, v 10.2).

Freshly thawed $A\beta_{1-42}$ in PBS with 1 mM EDTA was allowed to aggregate for 1.5-2 hours at room temperature at a concentration of 100 µM. Then, the oligomerized $A\beta_{1-42}$ solution was added to the recording solution to reach a final concentration of 500 nM $A\beta_{1-42}$. Once the recording protocol was performed in a slice in normal recording solution, the standard recording solution was switched to the same solution containing 500 nM $A\beta_{1-42}$. Slices were incubated for 20 minutes in this $A\beta_{1-42}$ containing solution before performing an identical recording protocol in the same patch-clamped cell.

1.2 Stereotaxic injection of kainic acid

The surgical procedure is identical compared to the ‘stereotaxic injection’ method described in the main manuscript (section 2.5) until the injection step. Here, 50 nL of 4 mg/ml KA dissolved in NaCl 0.9% or 50 nL NaCl 0.9% was injected into the DG (-2.20 AP, -1.40 ML, -2.10 DV). After injection, the syringe was left in place for 5 minutes, before slowly taking it out. The duration of anesthesia in this procedure ranged between 15 and 20 minutes.

1.3 Immunohistochemistry

We evaluated the correct location of the $A\beta_{1-42}$ injection with a 3,3’-diaminobenzidine (DAB) staining with an anti-amyloid β x-42 antibody (anti-12F4, Biolegend 803001) via a VECTASTAIN® Elite® ABC-HRP kit (Vector labs, PK-6102). After surgery, mice were deeply anesthetized with an overdose of 1.6 mg/g body weight pentobarbital (Dolethal, Vétoquinol). They were then transcardially perfused for 2 minutes with 0.9% NaCl, followed by perfusion with 4% paraformadehyde (PFA) in PBS for 5 minutes. Slices were postfixed in 4% PFA in PBS for 1 day and stored in TBS until slicing. We cut 40 µm coronal slices with a vibratome (Leica VT1000 S, Leica Biosystems) and these were stored in cryoprotectant medium (30% glycerol and 30% ethyleneglycol in TBS) at -20°C. For immunohistochemistry, slices at the injection location were selected and washed 3 times for 10 minutes in TBS. Then

we shielded the slices from light and incubated them for 30 minutes in 0.3% H_2O_2 (Merck KGaA, 1.07298.0250), after which they were put in blocking buffer (TBST [TBS with 0.1% Triton X] with 1% horse serum) for 1 hour. Subsequently, slices were incubated for 3 minutes in 80% formic acid followed by incubation overnight at 4°C with primary mouse anti-12F4 (1:1000). The next day, slices were incubated for 30 minutes in secondary biotinylated horse anti-mouse antibody (1:200, Vectastain, PK-6102), followed by incubation for 30 minutes in avidine-biotine complex (Vectastain, PK-6102). Between each step, slices were washed 3 times for 10 minutes in TBST. Slices were then put in acetate buffer (0.17 M sodium acetate in water at pH 6.0), followed by a staining in DAB-solution (0.05% DAB, 0.3% [v/v] HCl, 0.01 M ammoniumsulphate and 1 mM ammoniumchloride in acetate buffer) after which slices were washed twice in TBS. Slices were then mounted on slides, dehydrated in ascending alcohol series (30%, 70%, 95%, 100%; 5 minutes each) and put 3 times in Xylol for 5 minutes. Finally, slices were coverslipped with a few drops of DPX mounting medium (06522, Sigma Aldrich). Images were acquired with an EVOS Aperio microscope (GT450, Leica Biosystems).

Supplementary Table 1: overview of the methods used by Vande Vyver et al., Alcantara Gonzalez et al., Bellingacci et al., and Brouillette et al. for the intracerebral injection of Aβ1-42 oligomers and their seizure susceptibility (when applicable).

	Vande Vyver et al.	Alcantara Gonzalez et al.	Bellingacci et al.	Brouillette et al.
Dose	200 pmol in 1 µL per side (DG batch seizure after 1 week)<br>400 pmol in 2 µL per side (DG batch seizure after 90 min)<br>1 nmol in 10 µL (ICV)	1 nmol in 10 µL (ICV)	400 pmol in 4 µL unilateral (DG)	200 pmol in 2 µL per side (DG)
$A\beta_{1-42}$ solution	$A\beta_{1-42}$ oligomers in PBS with 1 mM EDTA	$A\beta_{1-42}$ oligomers in 2% DMSO and F12 medium	$A\beta_{1-42}$ oligomers in 2% DMSO and PBS	$A\beta_{1-42}$ oligomers in TBS with 1 mM EDTA
Validation of $A\beta_{1-42}$ species	TEM, ThT	/	/	TEM, Western blot
Control	Scrambled $A\beta_{1-42}$ in PBS + 1 mM EDTA	F12 medium	NaCl 0.9%	Scrambled $A\beta_{1-42}$ in TBS + 1 mM EDTA
Injection location	DG<br>ICV	ICV	DG	DG
Seizure model	i.v. PTZ/KA/4-AP	i.p. 4-AP	s.c. 4-AP and bicuculline	/
Outcome	No difference in seizure susceptibility	Increase in seizure susceptibility	Increase in seizure susceptibility	/

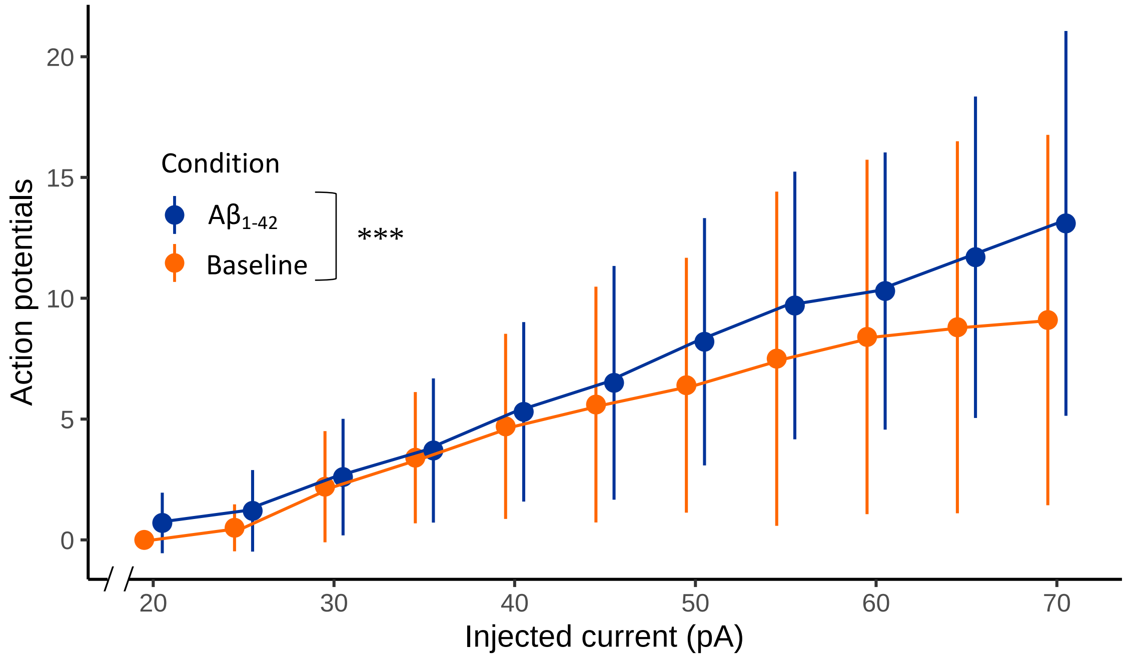

Supplementary Figure 1: We evaluated the effect of Aβ$_{1\text{-}42}$ oligomers on neuronal excitability of whole-cell patch-clamped dentate gyrus granular cells in acute hippocampal slices. First, the number of action potentials elicited in response to increasing injected currents was recorded in whole-cell patch clamped granular cells in normal perfusate. Then slices were incubated for 20 minutes in the same perfusate containing 500 nM Aβ$_{1\text{-}42}$ oligomers. Then, the same cells were again subjected to the same protocol of injected currents. An increase in current led to an increase in elicited action potentials ($F_{(1,207)} = 325$, $P < 0.001$). After incubation in Aβ$_{1\text{-}42}$ oligomers, more action potentials were elicited by a 70 pA current injection compared to baseline ($F_{(1,207)} = 17$, $P < 0.001$), meaning that neuronal excitability was increased after incubation with Aβ$_{1\text{-}42}$ oligomers. There was a significant interaction between the injected current and the condition, meaning that the difference between the groups increases with increasing current ($F_{(1,207)} = 6$, $P = 0.01$). A small horizontal shift per group was added to the data points to improve readability. Data are represented as mean ± standard deviation.

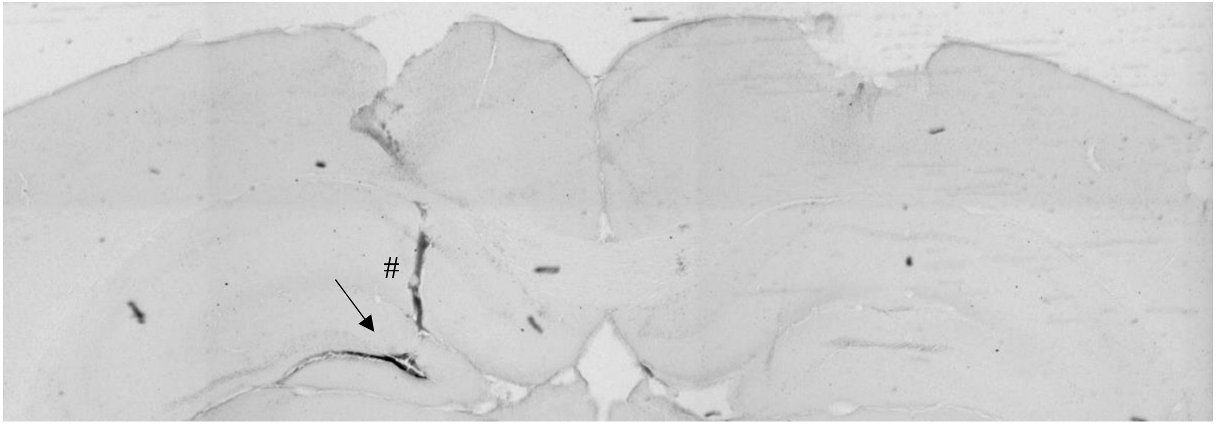

Supplementary Figure 2: Immunohistochemical validation of the successful injection of A$\beta_{1\text{-}42}$ in the DG. We performed a stereotaxic injection of 1 µL of A$\beta_{1\text{-}42}$ oligomers on the left and of 1 µL PBS 1 mM EDTA in right DG (AP: -2.2, ML: ±1.4, DV: -2.1). Fifteen minutes after injection, the mouse was perfused with 4% PFA. Slices were stained with an anti-A$\beta_{x\text{-}42}$ antibody using a DAB staining. This revealed the presence of A$\beta_{1\text{-}42}$ in the left, but not the right DG (indicated by the black arrow) and over the path of the needle (indicated by the black hash).

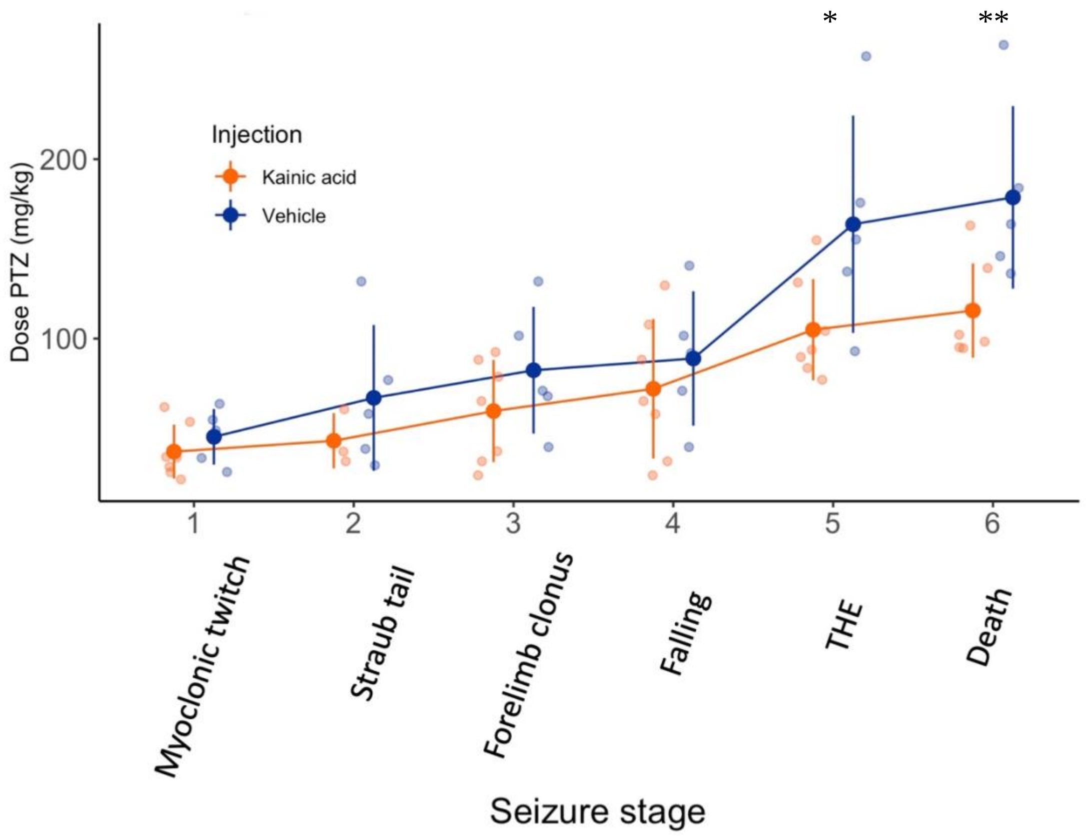

Supplementary Figure 3: We performed a single injection of 200 ng kainic acid or vehicle in the dentate gyrus of 7-week-old mice (n = 6 per group). One week after the intracerebral injection, mice were subjected to a continuous i.v. infusion of PTZ. This gave rise to progressively worsening seizures starting with a myoclonic twitch and eventually resulting in death ($F_{(5,46)} = 53$, $P < 0.001$). There is no difference in seizure susceptibility between groups at stage 1 ($F_{(1,10)} = 4$, $P = 0.09$), but there is an interaction between seizure stage and group ($F_{(5,46)} = 4$, $P = 0.005$). The post-hoc comparison showed that the mice who received kainic acid injection reached stage 5 (tonic hindlimb extension, THE) and 6 (death) more rapidly ($P = 0.01$ and $P = 0.006$ respectively). A small horizontal shift per group was added to the data points to improve readability. Data are represented as mean ± standard deviation. *p < .05 and **p < .01
